# Precise Measurements of the Absolute Nonlinear Refractive Index of Fused Silica Using High-Repetition-Rate Femtosecond Laser and Nonlinear Ellipse Rotation Signal

**DOI:** 10.1021/acsomega.6c04354

**Published:** 2026-08-04

**Authors:** Renato Mafra Moysés, João Victor Pereira Valverde, Renan Cunha, Cleber Renato Mendonça, Emerson Cristiano Barbano, Lino Misoguti

**Affiliations:** † Sao Carlos Institute of Physics, 117186 University of Sao Paulo (IFSC-USP), 13566-590, Sao Carlos, SP, Brazil; ‡ Departamento de Física, Instituto Tecnológico de Aeronáutica, 12228-900, Sao José dos Campos, SP, Brazil; § Physics Department, Federal University of Paraná, 81531-980, Curitiba, PR, Brazil

## Abstract

Here, we present a single-beam technique based on nonlinear ellipse rotation (NER) that, we believe, enables a more accurate determination of the absolute value of the electronic nonlinear refractive index of fused silica. We show that using high-repetition-rate femtosecond pulses enables NER to achieve a good trade-off among nonlinear signal, peak, and average laser power while remaining completely immune to thermal contributions. Also, NER can retrieve the laser pulse duration, linear chirp, and the nonlinear response under an identical experimental setup, ensuring fully self-consistent refractometry. We measured five commercial fused silica samples using ultrafast laser pulses with about 200 fs, at three wavelengths: 343, 514, and 1028 nm, reinforcing its role as the metrological benchmark for next-generation photonic materials and devices. All tested fused silica samples showed no significant differences in their nonlinear refractive properties, yielding *n*_2_ = (1.9 ± 0.3) × 10^–20^ m^2^/W at 1028 nm, *n*_2_ = (2.3 ± 0.3) × 10^–20^ m^2^/W at 514 nm, and *n*_2_ = (2.7 ± 0.5) × 10^–20^ m^2^/W at 343 nm. All values are consistent with those reported in the literature but slightly smaller than those usually accepted as references.

## Graphical abstract

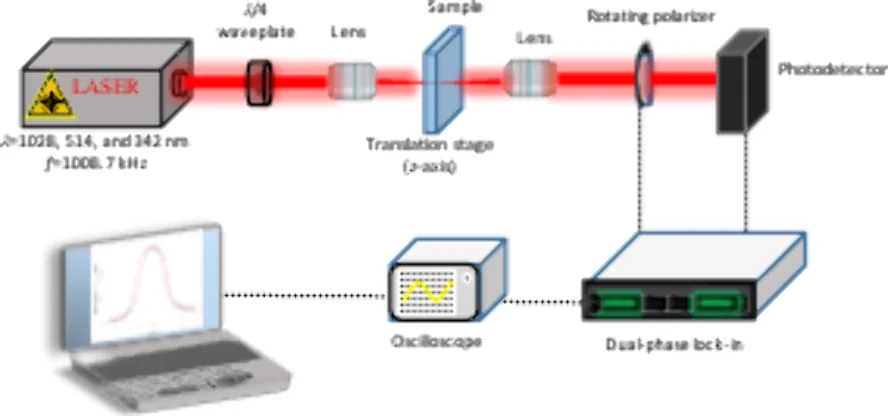

## Introduction

Advances in modern photonics materials production have intensified the demand for determining nonlinear optical parameters, such as the ultrafast third-order nonlinear refractive index (*n*_2_). New photonic media, such as two-dimensional materials,^1^ thin films,^2^ and nanoparticles,^3^ require precise *n*_2_ values to determine whether they have potential for device applications. Even a small uncertainty can propagate into large deviations in the modeling and designing of ultrafast photonic devices, such as all-optical switches,^4^ for instance. This challenge is particularly evident in the determination of *n*_2_ values, since most measurements rely on comparison with a well-known reference material. However, even for one of the best and widely used reference materials, fused silica (FS), the *n*_2_ value and dispersion are still not well-established.

FS is one of the most important materials used as a reference for *n*_2_ value and plays a central role in modern photonics, forming the backbone of optical fiber communication networks and serving as a key platform for integrated components, microresonators, and ultrafast nonlinear devices. Accurate knowledge of its third-order nonlinear refractive index is essential for predicting and controlling nonlinear processes such as self-phase modulation, all-optical switching, and pulse propagation in both bulk and waveguide geometries. Nevertheless, despite decades of study, reported *n*_2_ values for fused silica exhibit significant discrepancies, reflecting the intrinsic challenges of achieving absolute, thermally independent, and self-consistent measurements. FS is widely established as a reference in several studies due to its simple chemical structure, great mechanical and thermal stability, and relatively low manufacturing cost. In addition, it exhibits a smooth refractive index dispersion curve,^5^ without major changes over wavelength, isotropic symmetry (no second-order effects), and a wide transparency band from the ultraviolet to the near-infrared, with low linear and nonlinear absorption in the visible region.^6^ Several studies have already been carried out to determine the *n*_2_ value of silica for different wavelengths, to reliably tabulate the reference value on which the other materials should be based. Moreover, differences in *n*_2_ values between FS’s from different manufacturers are present in the literature,^7^ indicating that small variations in the density of the sample, in the transmittance spectra,^6^ or in the linear refractive index could result in considerable differences in *n*_2_.

On the other hand, direct measurement of the absolute value of *n*_2_ is not straightforward due to several issues. Numerous techniques developed for precise and sensitive measurements of *n*_2_, such as optical Kerr effect in a waveguide,^8^ pump–probe,^7^ interferometry,^9,10^ far field intensity profile change,^11^ closed-aperture *Z*-scan,^12,13^ etc., have drawbacks that prevent good absolute value determination. For example, two or more beam methods suffer from beam overlap and phase-matching problems. *Z*-scan is a single-beam method that has no phase-matching or beam alignment issues but is sensitive to multiple distinct nonlinear refractions, including the cumulative thermal one, which need to be discriminated. Furthermore, *Z*-scan is very dependent on the transversal laser mode quality (Gaussian mode) and sample quality (surface and bulk scattering, for instance). In this way, a large discrepancy between the values reported in the literature is observed,^14^ along with high inaccuracies presented by the different techniques applied. Measurements performed at a high-repetition rate of laser pulses can lead to unreliable values of *n*_2_, since thermal effects arise from interpulse heating even when femtosecond pulses are applied.^15^ Furthermore, long pulses (in the order of pico and nanoseconds) can lead to noninstantaneous nuclear contributions^16–18^ and hence hinder the true ultrafast *n*_2_. In addition, most of the measurements in the literature are performed with low-repetition, high-power, ultrafast amplified laser systems (kHz) not only to avoid cumulative nonlinear effects but also due to their broad commercial availability and a good wavelength tuning range when associated with a nonlinear conversion system (ex: optical parametric amplifiers (OPA)). But, in this case, due to high-peak power, only pulses with a few nanojoules are required for most of the measurements, independently of the experimental method and setup, and, in this way, the required laser average power (*P*_*avg*_) is quite low and hard to measure with a conventional power meter with a thermal sensor. Finally, besides the pulse energy and laser beam parameters, to determine the laser irradiance in the sample, it is necessary to measure the pulse width (τ). Indeed, there are several methods appropriate for determining τ, such as FROG,^19^ SPIDER,^20^ or MIIPIS.^21^ Although very precise and sophisticated, such measurements require separate devices and can measure different pulse widths and dispersion due to a distinct pulse propagation path.

To overcome, or at least reduce these problems, here we propose to measure the absolute value of the pure electronic *n*_2_ of FS using the nonlinear ellipse rotation (NER)^22–24^ effect and high-repetition-rate ultrafast laser pulses. The NER measurement is particularly attractive among other nonlinear signals, mainly for four reasons: first, although the use of high-repetition-rate (MHz) laser sources may lead to cumulative thermal effects that can distort the intrinsic electronic response of a material,^25^ NER is insensitive to thermal effects^26^ and can be employed in these cases; second, it is a single-beam measurement, and no phase-matching or beam-overlap problems occur;^24^ third, it is possible to determine both the *n*_2_ and pulse width τ, using the same experimental setup;^22,23^ and, finally, using a dual-phase lock-in, NER can be measured as a phase signal,^22,24^ not as magnitude,^27^ and, in this way, it is much less sensitive to optical sample quality and light intensity fluctuations. In addition, a MHz-repetition-rate laser enables more accurate average power measurements and, consequently, significantly reduces uncertainty in laser irradiance compared with kHz laser systems. To test our method and provide reliable *n*_2_ reference values to the literature, we characterize five different commercial FS samples, Schott SQ1, Herasil, Suprasil, Corning 7980, and Infrasil, in three different wavelengths: 1028, 514, and 343 nm to give the dispersion information.

## Methods

### Nonlinear Ellipse Rotation (NER)

NER^24^ is a Kerr-type effect that occurs when an elliptically polarized laser beam propagates through a nonlinear medium, causing the elliptical polarization to rotate. This rotation is directly proportional to the *n*_2_ of the sample, according to *n = n*_0_ *+ n*_2_ *I,* where *n* is the total refractive index, *n*_0_ is the linear refractive index, and *I* is the irradiance, and thus is directly related to the third-order nonlinear electrical susceptibility tensor χ.^3^ Taking into consideration the Kerr effect in isotropic samples, χ^(3)^ can be simplified into χ^3^ = *A/3* + *B/6*, where *A = 6 χ* ^(3)^_1122_ and *B* = 6 χ^(3)^_1221_ ^24^ are the only independent nonzero elements from the tensor, with *B = A* for nonlinear refraction (NLR) originated from nonresonant electronic effects, *B = 6A* for molecular orientation (nuclear) effects, and *B = 0* for thermal effects.^28^ Since the elliptical polarization rotation depends only on *B*, NER measurements are intrinsically insensitive to thermal effects.^29^ Hence, in this case, the pure electronic *n*_2_ can be directly written as

1 $$n_{2}=\frac{3B}{8n_{0}^{2}\varepsilon_{0}c}$$

The measurements are performed via a phase-sensitive method to obtain the ellipse rotation angle (α), by associating it with phase shifts in the signal caused by the nonlinear refractive effect, while the sample is propagated along the focus of the laser beam, and converting them into *n*_2_. According to previous works,^22,24^ this angle is described by the following equation:

2 $${\left\langle \alpha \left(z\right)\right\rangle }_{LIA}=\frac{1}{\sqrt{2}}\frac{2\pi }{\lambda }\frac{\sin\left(2\varphi \right)}{2}\frac{B}{4n_{0}^{2}\varepsilon_{0}c}\left(n_{0}z_{0}\right)I_{0}\left[\tan^{-1}\left(\frac{z_{B}}{z_{0}}\right)-\tan^{-1}\left(\frac{z_{A}}{z_{0}}\right)\right]$$

where *z* is the position of the sample over the *z-*axis, λ is the laser wavelength, φ is the angle of the quarter-wave plate used to generate elliptical polarization, *ε*_0_ and *c* are the electric permittivity and speed of light in vacuum, respectively, *z*_0_ *= πw*_0_^2^/λ is the Rayleigh range, *w*_0_ is the Gaussian beam waist, *z*_*B*_ = *z* + *t*/(2*n*_0_) and *z*_*A*_ = *z* – *t*/(2*n*_0_) are the effective sample displacements in which *t* is the sample thickness, and the arctangent functions are related to the focalization regime. In this case, a phase-sensitive method can rely on a dual-phase lock-in amplifier (LIA) and a rotating polarizer, which, along with the assumption of ultrafast, temporally and spatially Gaussian beams, yields a 1/√2 factor. The LIA can then interpret the rotation α as a phase shift between the signal with and without the influence of the NLR.

One of the most useful aspects of this technique is that it can be performed under a strong focalization regime (*t ≫ n*_0_ *z*_0_), making it possible to map the effect of the NLR as the pulse propagates through the sample. This feature can be used to determine the linear chirp and the temporal duration τ of the laser pulse,^23^ without the need to change the experimental setup used to determine *n*_2_. This is due to the stretching or shortening of the pulse as it propagates through the sample, which can be understood by considering that, for a considerably long optical path, τ is affected by the relationship between the sample’s group velocity dispersion (GVD) and the pulse initial group delay dispersion (GDD). Since the laser peak intensity depends on the pulse duration, through *I*_0_(τ) = [4√ln(2)]*P*_*avg*_/(π^3/2^ *w*_0_^2^ *τΓ*_*R*_), where Γ_*R*_ and *P*_*avg*_ are the laser repetition rate and average power, respectively, and the ellipse rotation angle depends on *I*_0_, eq (2) also becomes a function of pulse duration, i.e., α → α­(*τ,z*). Hence, defining *L* (∝*n*_0_ *z*) as the focal point distance relative to the sample input interface, we can write^30^

3 $$\tau \left(L\right)=\tau_{0}\sqrt{1+{\left[\frac{4\ln2D_{2}\left(L\right)}{\tau_{0}^{2}}\right]}^{2}}$$

Substituing τ­(*L*) into the *I*_0_ term of eq (2) then makes it possible to determine both the pulse chirp *D*_2_ and duration τ. In eq (3), *D*_2_ *(L) = GDD + GVD* × *L* is the pulse chirp as a function of propagation and τ_0_ is the transform-limited (TL) pulse duration, which we assume to be Gaussian and so τ_0_ = 0.44λ_0_^2^/(*c*Δλ),^30^ where λ_0_ is the central wavelength and Δλ is the spectral bandwidth of the laser determined at full width at half-maximum (fwhm), which can be measured with a spectrophotometer.

This dual determination is made by taking the experimental data obtained by *<α­(z)>*_*LIA*_ and adjusting the parameters in eq (2) until the fitting matches the data. The interesting point of this procedure is that it is not always necessary to know either the sample’s *n*_2_ or the average laser power to obtain *D*_2_ and τ, since *τ­(L)* usually provides a unique shape to *<α­(z)>*_*LIA*_, while the other parameters only adjust its value.^23^ Indeed, this only happens if the sample is thick enough (in comparison to the focal regime) or has high enough GVD so that *τ­(L)* affects α sufficiently to the point of the choice of *D*_2_ and τ becomes unique in the fitting, independently of the value provided by *φ, P*_*avg*_, and *B*. Nevertheless, the limitation is that the sample’s GVD must always be well-known and preferably high.

Unfortunately, FS has a small GVD in the visible and IR ranges and cannot be used for pulse-width measurement with NER. Therefore, for visible and IR, the method proposed here to determine *n*_2_ should follow two steps: first, we have to evaluate the pulse width using a thick sample with high GVD, such as SF6 for 1028 nm and LaSF-N30 for 514 nm, and then, measure FS itself (can be a thin sample) with the same setup previously calibrated. To measure the UV (343 nm) pulse width, due to the strong absorption of SF6 and LaSF-N30, we must use a very thick FS sample (38.5 mm) to compensate for its low GVD.

The high-repetition rate of the laser will ensure accurate average laser power measurement and, in turn, precise pulse energy determination. Finally, one last uncertainty is the ellipticity state of the laser beam, which can be reduced by measuring the NER signal as a function of the angle of the quarter-wave plate as predicted by eq (2).

### Experimental Setup

The experimental apparatus is shown in Figure 1. It consists of a laser system (Carbide model, *Light Conversion*, *Inc.*) operating at 1028, 514, and 343 nm with pulse durations in the order of hundreds of femtoseconds. According to the manufacturer’s manual, for 1028 nm (fundamental), the duration of the TL pulse is in the order of 216 fs. This laser can generate pulses at 1008.7 kHz for the three wavelengths cited with a well-behaved Gaussian mode, providing stable energies in the order of a few nanojoules for a good NER signal and, consequently, at the mW level of power. The average power at each of the three wavelengths is measured using a power meter (StarLite model, *OPHIR*), which enables *P*_*avg*_ to be determined with an error of no more than 5%, if mW power levels are used.

**Figure 1:**
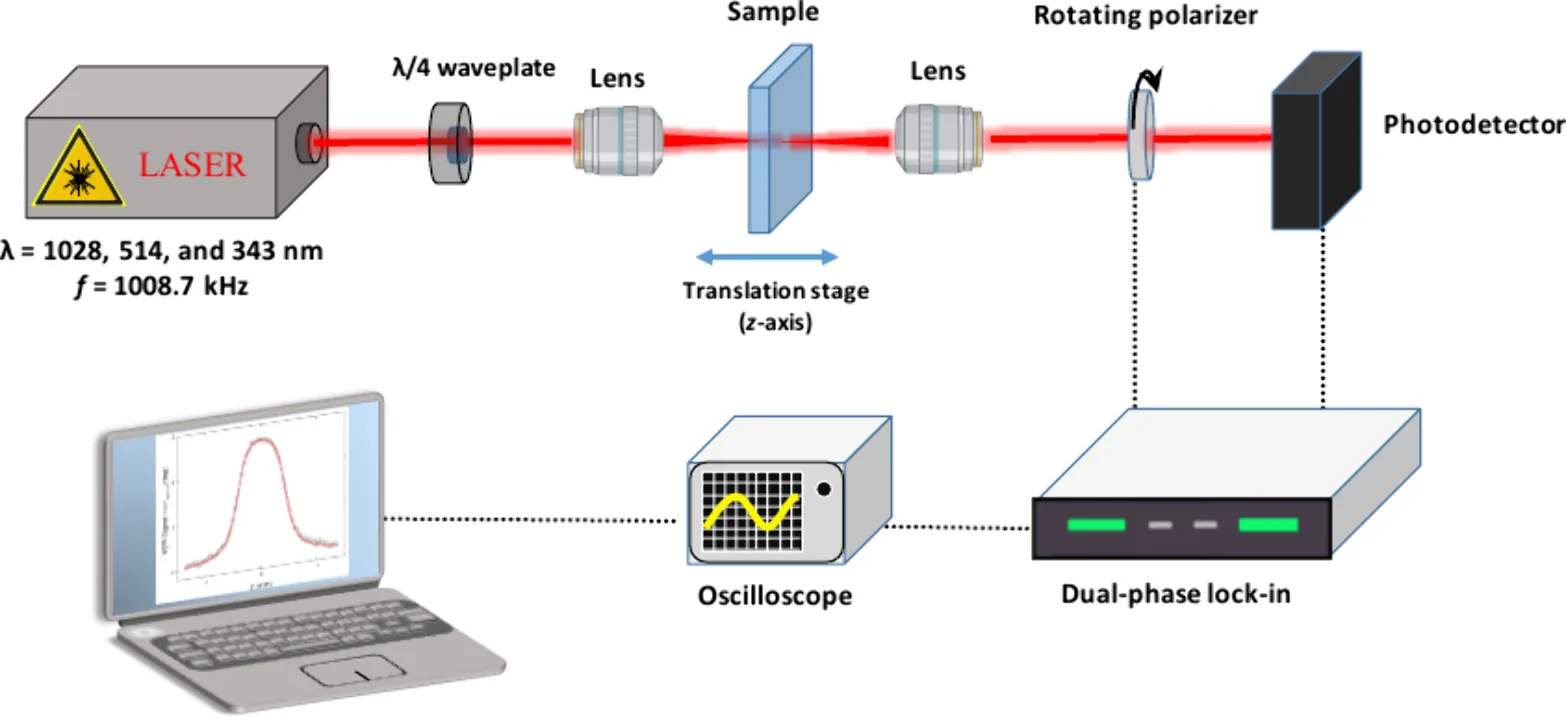
Diagram of the experimental setup employed for NER measurements. A femtosecond Yb:KGW laser system (λ = 1028, 514, and 343 nm) is operated at high laser pulse repetition rate (*f* = 1008.7 kHz) to characterize the nonlinear refractive signal of a moving sample (*z*-axis translation) with the use of a pair of lenses, a quarter-waveplate, a rotating polarizer, a dual phase lock-in amplifier (LIA), a silicon photodetector, and a digital oscilloscope coupled to a computer.

The laser beam passes through an appropriate quarter-waveplate (λ/4) to generate elliptical polarization, crucial for NER. Here, it is important to point out that linear polarization is achieved when the waveplate is set to φ = 0°, 90°, 180°, ..., and no NER signal is observed. For pulse width measurement, the magnitude of the NER signal is not crucial; therefore, we have worked with fixed φ = ∼ 22.5°, since it provides the best signal-to-noise-ratio.^22^ On the other hand, to measure the absolute magnitude of *n*_2_ of FS, we fully changed the polarization state from linear to circular (right and left) to remove any uncertainty about the polarization state of the laser beam.

For all measurements, the typical rotation frequency of the linear polarizer was ∼ 40 Hz. To measure the NER signal, we used a commercial LIA (7225 DSP Lock-in Amplifier model, SIGNAL RECOVERY). In the open-configuration, most of the light transmitted through the second lens is collected by a Si-based photodetector (DET100A/M model, *ThorLabs*: 400–1100 nm).

The laser spectral bandwidth is measured by a portable spectrometer (HR4000 model, *Ocean Optics*: 200–1100 nm, 5 μm slit, 0.75 nm fwhm resolution). To determine the pulse duration at 1028 and 514 nm, respectively, two optical calibration glasses from the Schott catalogue, SF6 (thickness *t* = 12.4 mm) and LaSF-N30 (*t* = 12.2 mm), are chosen. For 343 nm, we must use a long FS (*t* = 38.5 mm). Their linear refractive index and group velocity dispersion are already well-characterized^5,31,32^ and summarized in Table 1. For pulse-width measurement, it is not important to know the *n*_2_, but its values can be found in the literature. For example, for SF6 at 1028 nm *n*_2_ ∼ 1.61 × 10^–19^ m^2^/W.^33^ To validate our values of pulse duration, intensity autocorrelation (IAC) measurements via second-harmonic generation (SHG) are performed with a commercial device (GECO Scanning Autocorrelator model, *Light Conversion*, *Inc.*, equipped with BBO crystals of 0.02 mm thickness for the 500–1050 nm range and 0.1 mm thickness for the 1000–2000 nm range, a Si-based photodetector, and a measurement resolution of 10–20000 fs).

**Table 1:** Thick Sample Properties for the Pulse Width Measurements by NER^a^

**Sample**	**Thickness (mm)**	**Wavelength (nm)**	***n***_**0**_	**GVD (fs** ^**2**^ **/mm)**
**SF6**	12.4	1028	1.7747	136.92
**LaSF-N30**	12.2	514	1.8121	197.09
**Silica**	38.5	343	1.4781	123.54

a Literature values for *n*_*0*_ and GVD were used: SF6,^32^ LaSF-N30,^31^ and Silica.^5^

In the experimental setup, for λ = 1028 and 514 nm, we have used a regular 4× microscopy objective lens (*f* ∼ 3 cm) to provide a strong (not so strong) focalization regime for SF6 and LaSF-N30 (all thinner FS samples). For λ = 343 nm, we need to use FS lenses, and due to the long sample (38.5 mm), an *f* ∼ 15 cm could provide a thick condition for pulse width measurement. The five distinct commercial FS samples characterized have a thickness of approximately 1.25 mm and a diameter of 25.4 mm.

## Results and Discussion

### Pulse Width Determination with NER

First, we determined the laser pulse bandwidth at 1028, 514, and 343 nm to predict the TL pulse width. Figure 2 shows the normalized spectra of the three laser wavelengths measured with a spectrophotometer (points) and Gaussian fitting (line) (Table 2). For 1028 nm, we observed an unexpected result. Basically, the experimentally measured bandwidth (8.9 nm) was wider than that reported by the manufacturer (7.2 nm), which implies shorter pulses. We believe that this large bandwidth measured is due to the influence of crystal fluorescence background, and it is not related to the true ultrashort-pulse bandwidth. According to the laser manual, the pulse width limit is around 220 fs, and the correct pulse bandwidth is 7.2 nm.

**Figure 2:**
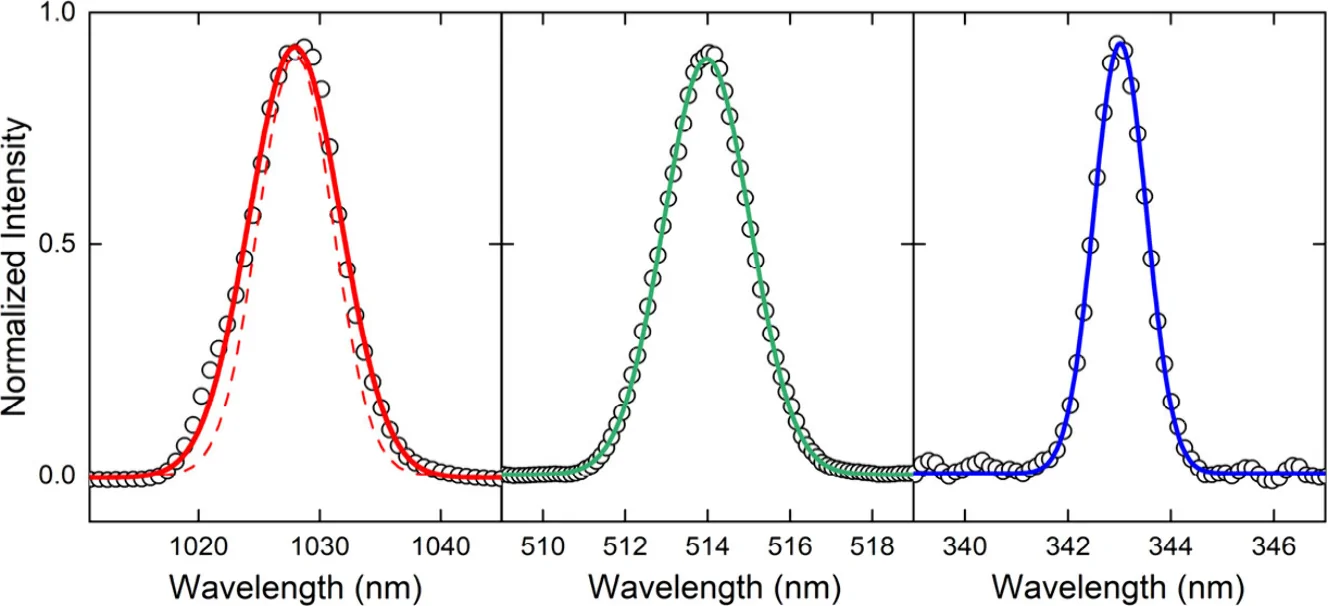
Laser spectra (black circles) at 1028 nm (fundamental), 514 nm (second harmonic), and 343 nm (third harmonic). The solid red, green, and blue curves correspond to Gaussian fits of the fundamental, second, and third harmonics, respectively. The red dashed curve represents a Gaussian fit obtained from the manufacturer’s specified spectral width.

**Table 2:** Pulse Properties Determined with NER Measurements and GECO SHG-IAC

**Wavelength (nm)**	**Bandwidth, Δ** ***λ*** **(nm)**	**TL pulse width,** ***τ***_**0**_ **(fs)**	**Input pulse width with NER,** ***τ*** **(fs)**	**Pulse width (GECO),** ***τ*** **(fs)**	**Pulse GDD with NER (×1000 fs** ^**2**^ **)**
**1028**	7.2 ± 0.9	220 ± 30	250 ± 20	249	10 ± 2
**514**	2.5 ± 0.3	150 ± 20	180 ± 20	181	5 ± 1
**343**	1.2 ± 0.2	140 ± 30	160 ± 20	-	3 ± 1

Nevertheless, from a practical point of view, both bandwidths yield about the same pulse duration in SF6 glass (Figure 3a) in the NER measurements. But, in fact, we assumed Δλ = 7.2 nm, the fitting (red dashed curve) (Figure 2) which gives us a better match to the experimental data.

**Figure 3:**
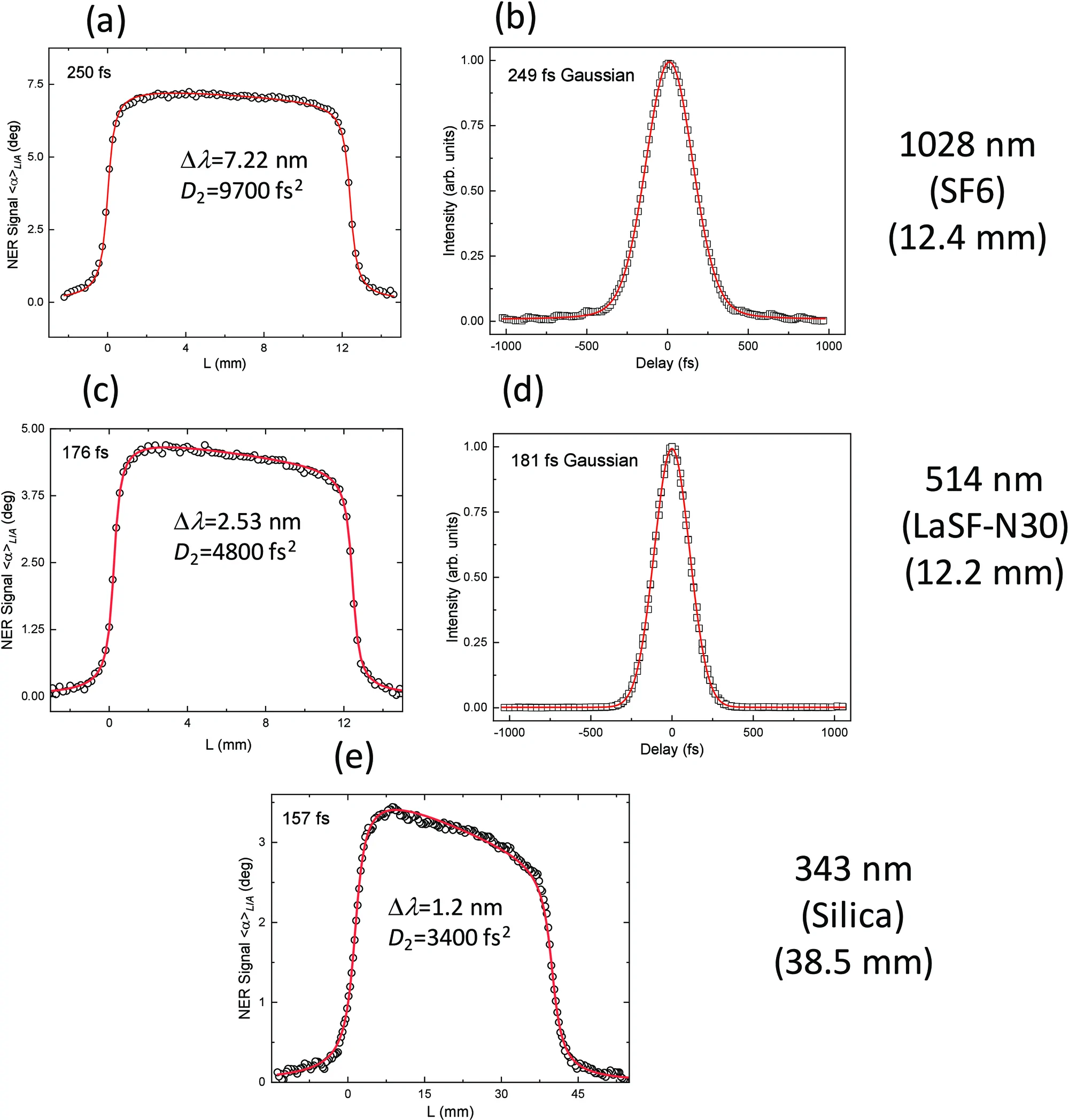
NER measurement in SF6, LaSF-N30 and Silica at 1028, 514, and 342 nm, respectively, to determine the pulse temporal duration, black circles represent the experimental data, and the red curves are fittings using eqs 2–3) with respective Δλ and *D*_2_. GECO SHG-IAC measurement with a Gaussian fit for 1028 nm (b) and 514 nm (d). GECO SHG-IAC measurements are not possible at 343 nm.

The measurement at 1028 nm shown in Figure 3(a) was obtained with laser *P*_*avg*_ = (20 ± 1) mW (@ ∼ 1 MHz). Although we have set to the shortest pulse width (TL τ_0_ ∼ 220 fs) in the Carbide laser, the NER result of τ­(*L* = 0) = (250 ± 30) fs and ∼ 9700 fs^2^ GDD. The pulse width is consistent with the one obtained by the autocorrelator (Figure 3(b)). Moreover, even though NER and GECO measurements are performed with different optical paths, at 1028 nm, we do not expect optical elements (such as mirrors) to present considerable GVD in such a way as to result in a significant difference in the pulse durations. This result indicates that there is a small offset in the built-in pulse compressor position for TL pulses.

For the second harmonic, we performed NER measurements in LaSF-N30 (Figure 3(c). The average power employed was *P*_*avg*_ = (4.0 ± 0.2) mW (@ ∼ 1 MHz). The experimental data (black circles) were fitted with our model (red curve), yielding the pulse parameters given in Table 2.

Moreover, from GECO, we found τ = 181 fs (Figure 3(c)), which is very close to the values obtained by NER (180 fs) and to the expected value for SHG.^34^

As mentioned, for the third harmonic, we need to use another lens with a long focal length and a long sample. We have used *P*_*avg*_ = (6.5 ± 0.3) mW, (@ ∼ 1 MHz). As seen in Figure 3(e), we have only NER measurement since GECO cannot measure this wavelength. The results obtained with NER for 343 nm are summarized in Table 2.

Even using relatively low laser power, we observed a small photodarkening and color-center formation in FS. Nevertheless, we couldńt see much effect in the pulse width determination. Repetitively, we observe the same pulse width in all measurements.

### Nonlinear Refractive Index Determination with NER

Once the pulse properties were determined, we performed NER measurements in the five thin commercial FS samples. For each FS, we measured NER as a function of the laser beam ellipticity by adjusting the λ/4 waveplate angle (∼10 ° displacement). For λ_0_ = 1028 nm, the average power employed was *P*_*avg*_ = (34 ± 2) mW (@ ∼ 1 MHz, τ = (250 ± 20) fs). As an example, we can see some measurements in Infrasil, Figure 4(a), where black circles are the experimental data and the red curves are the fitting using eq 2–3) with all the same parameters except the waveplate angle φ.

**Figure 4:**
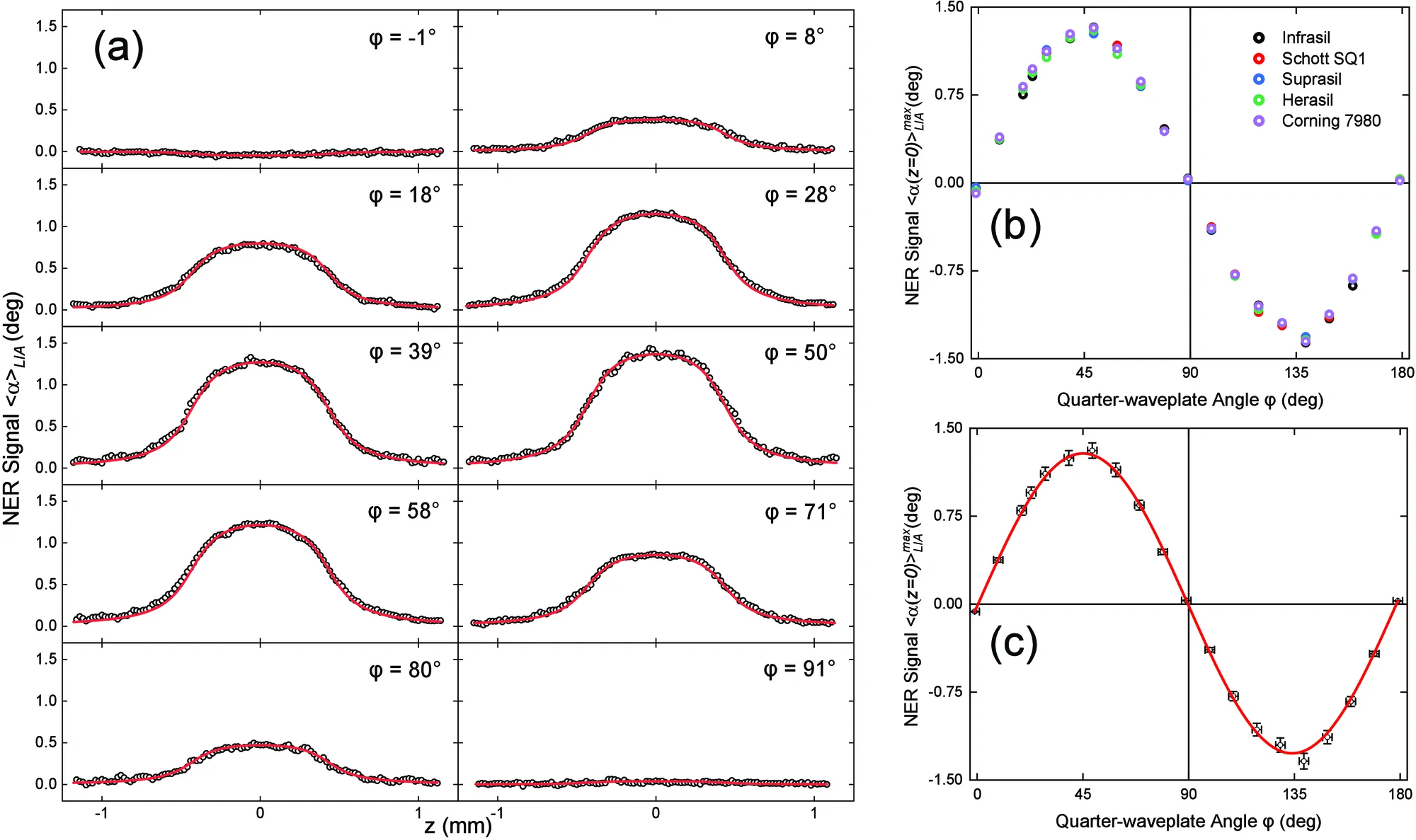
(a) Some of the NER signal measurements performed in Infrasil at λ = 1028 nm as a function of the laser polarization. The black circles represent experimental data of <α>_LIA_ as a function of sample propagation on the *z*-axis, and the red curves are theoretical fittings using eqs 2–3). (b) Maximum values (sample at focus) of the ellipse rotation <α­(*z* = 0)>^max^_LIA_ obtained by NER measurements for each of the five FS samples as a function of the waveplate angle *φ.* (c) Mean value of <α­(*z* = 0)>^max^_LIA_ among the five samples calculated (black circles) and sine fitting according to eq 2–3) (red curve).

The maximum NER signals for each FS as a function of the waveplate angle are plotted in Figure 4(b). The five silicas did not demonstrate significant differences, and the experimental dispersion does not exceed 5%. In Figure 4(c), we display the averaged values and the *sin*(2φ) dependence, consistent with eq 2–3). The error bars in the experimental data in Figure 4(c) come from the dispersion among the five samples.

Thus, by accounting for all uncertainties, it was possible to determine *n*_2_^*FS, λ=1028 nm*^ = (1.9 ± 0.3) × 10^–20^ m^2^/W. This value is similar to the ones obtained by Boudebs et al.,^35^ Shimada et al.,^13^ DeSalvo et al.,^12^ and Schiek.^36^

For λ_0_ = 514 nm, we performed the same NER measurements as for 1028 nm; for all five FS, using *P*_*avg*_ = (9.0 ± 0.5) mW (@ ∼ 1 MHz, τ = (180 ± 20) fs). As can be seen for SQ1 (Figure 5(a)), similar behavior was observed.

**Figure 5:**
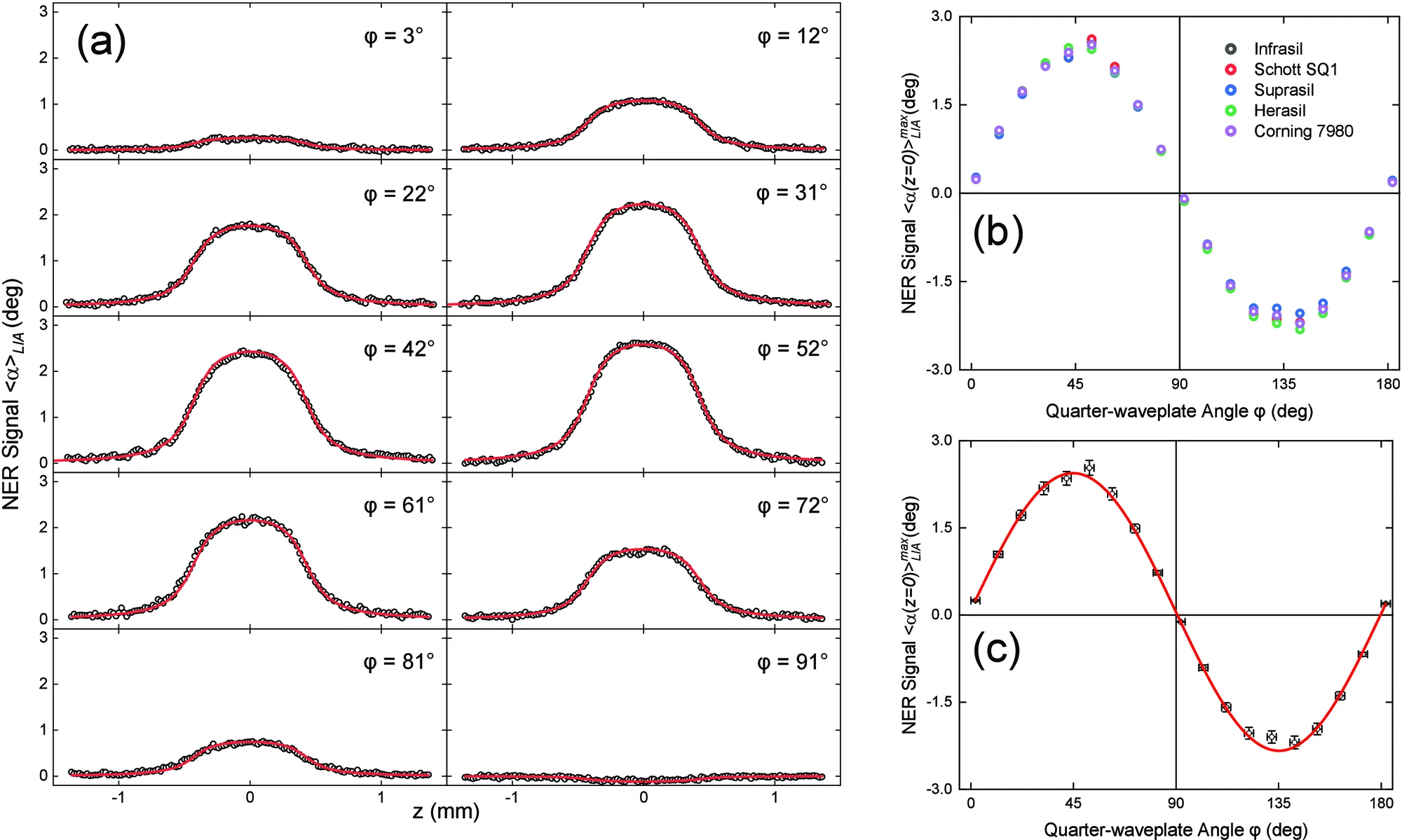
(a) Some of the NER measurements performed in Schott SQ1 at λ = 514 nm as a function of the laser polarization. The black circles represent experimental data of <α>_LIA_ as a function of the sample propagation on the *z*-axis, and the red curves are theoretical fittings using eq 2–3). (b) Maximum values (sample at focus) of the ellipse rotation < α­(*z* = 0)>^max^_LIA_ obtained by NER measurements for each of the five FS samples as a function of the waveplate angle φ. (c) Mean value of <α­(*z* = 0)>^max^_LIA_ among the five samples calculated (black circles) and sine function fitting (red curve).

In Figure 5(b), we can see all the measurements for the five FS samples. In Figure 5(c), we show the mean value from all data (black circles) and the theoretical fitting (eq 2–3)) to calculate the *n*_2_ values.

According to the experimental data, the nonlinear refractive index for FS is *n*_2_^*FS, λ=514 nm*^ = (2.3 ± 0.3) × 10^–20^ m^2^/W. This is equivalent to the results obtained by DeSalvo et al.,^12^ in which it was found *n*_2_ = (2.24 ± 0.46) × 10^–20^ m^2^/W at λ = 532 nm.

Lastly, we performed measurements in the third harmonic (λ_0_ = 343 nm) using *P*_*avg*_ = (6.5 ± 0.3) mW (@ ∼ 1 MHz, τ = (160 ± 20) fs) for all five FS. Figure 5(a) shows some of the measurements for Corning 7980. In contrast to the other two previous wavelengths, in this case, a clear thin-sample condition is observed, since a *f* = 15 cm lens was used.

Again, there is no significant difference in the ellipse rotation angles found for the samples (Figure 6(b). The mean value is calculated from the samples and can be visualized in Figure 6(c), in which the experimental data (black circles) and theoretical fit, eq 2–3 (red line), yield the best NLR value.

**Figure 6:**
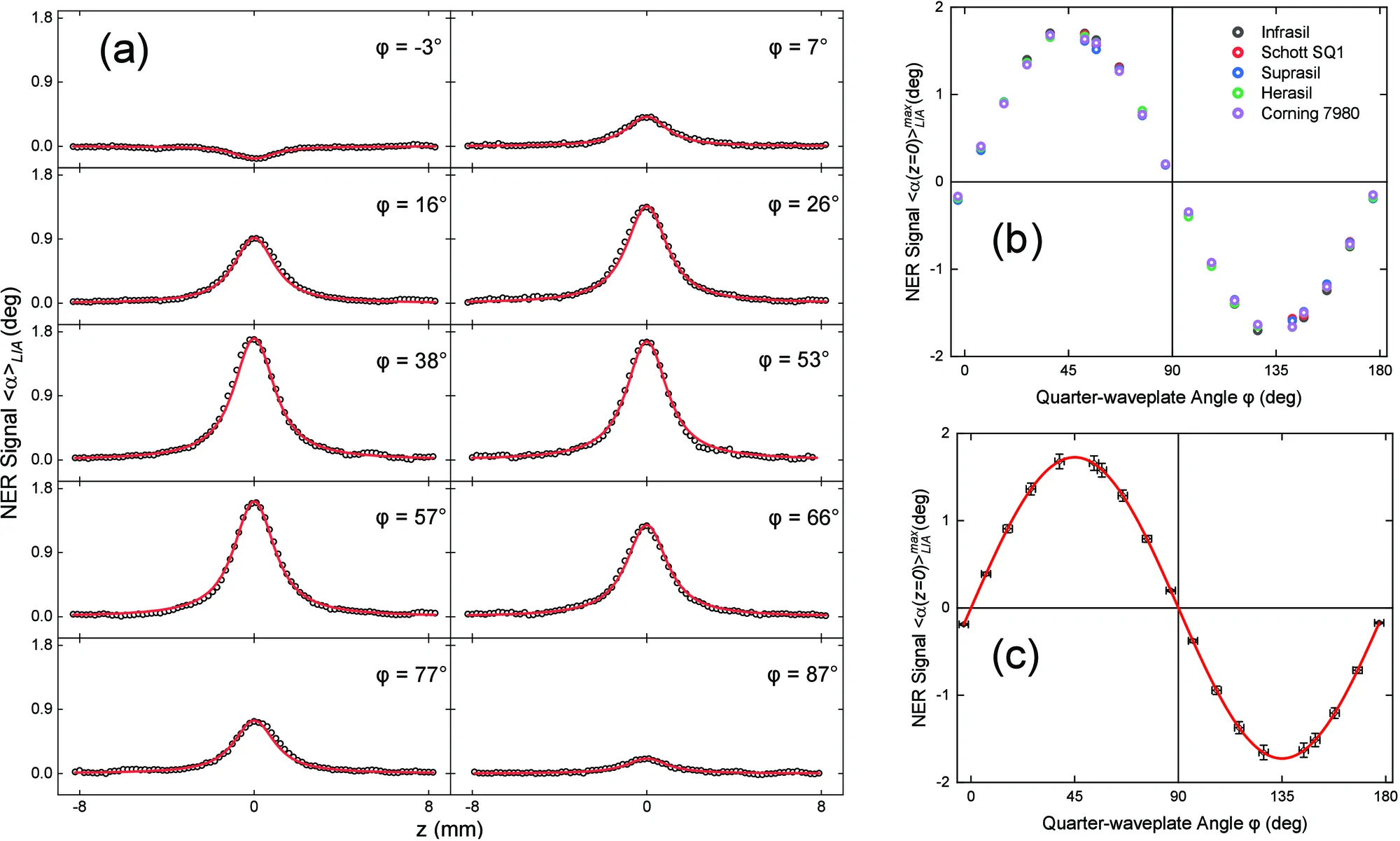
(a) Some of the NER measurements performed in Corning 7980 at λ = 343 nm as a function of the laser polarization. The black circles represent experimental data of <α>_LIA_ as a function of the sample propagation on the *z*-axis, and the red curves are theoretical fittings using eqs 2–3). (b) Maximum values (sample at focus) of the ellipse rotation <α­(*z* = 0)>^max^_LIA_ at λ = 343 nm obtained by NER measurements for each of the five FS samples (different colored circles) as a function of the waveplate angle φ. (c) Mean value of <α­(*z* = 0)>^max^_LIA_ among the five samples calculated (black circles) and sine function fitting (red curve).

The best value found for FS at λ = 343 nm was *n*_2_^*FS*^ = (2.7 ± 0.5) × 10^–20^ m^2^/W. This result is similar to that reported by Kim et al.,^11^ who found *n*_2_ = (3.00 ± 0.22) × 10^–20^ m^2^/W at λ = 308 nm.

As mentioned before, there is a significant dispersion among measurements published in the literature, so we can always find results similar to those obtained here. However, it is important to highlight that the published average values are slightly higher than our results are summarized in Table 3. To illustrate this, we also plotted data from the literature. In Figure 7(a), in which it is possible to visualize different values of *n*_2_ (colored circles) for the region of λ between 308 and 1550 nm, varying approximately from 1.8 up to 3.0 × 10^–20^ m^2^ */*W. Additionally, using the compiled data by D. Milam,^14^ including the determination of a dispersion curve (red curve in Figure 7(b)), our results (black circles) exhibit the same dispersion. Still, they are approximately 27% lower than the Milam curve.

**Table 3:** Nonlinear Refractive Index of FS Determined with NER Measurements Using a High-Repetition-Rate Laser

**Wavelength (nm)**	***w***_***0***_ **(μm)**	***n***_**2**_ **(×10** ^**–20**^ **m** ^**2**^ **/W)**
**1028**	7.0 ± 0.4	1.9 ± 0.3
**514**	4.8 ± 0.2	2.3 ± 0.3
**343**	10.6 ± 0.5	2.7 ± 0.5

**Figure 7:**
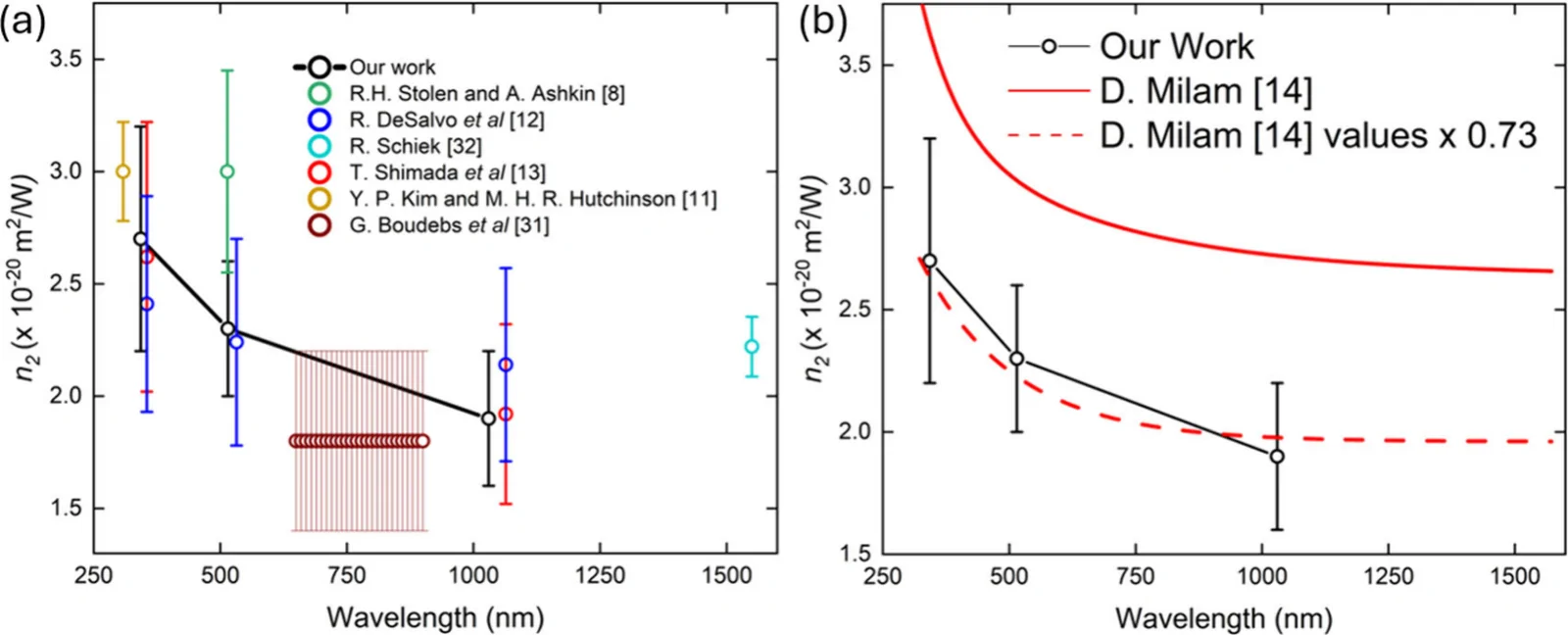
(a) Some of the fused silica nonlinear refractive index (*n*_2_) values available in the literature (colored circles) compared to the ones obtained by this work, as a function of laser wavelength. (b) The obtained *n*_2_ values by this work (black circles) as a function of wavelength compared to the well-established D. Milam^14^ proposed dispersion curve (red continuous curve) for fused silica. For comparison, our results are approximately 27% lower (red dashed curve) but follow similar dispersion behavior. The black lines connecting our results are shown merely to guide the eye.

## Conclusion

In summary, we propose a precision refractometry technique based on NER with ultrafast high-repetition-rate pulses to determine the absolute nonlinear refractive index of materials. This method allows the determination of the laser pulse characteristics (linear chirp and temporal duration) in the same experimental setup and conditions in which the nonlinear refraction signal is obtained, the precise determination of the average power, the minimization of noise in the measurements due to the operation in strong focalization regime with high-repetition-rate pulses, and hence, the obtention of reliable values of *n*_2_ at distinct wavelengths.

To validate our method, the nonlinear optical refractive properties of five distinct commercial FS samples (Schott SQ1, Corning 7980, Herasil, Infrasil, and Suprasil) were characterized at the wavelengths of 1028, 514, and 343 nm. The *n*_2_ results obtained were (1.9 ± 0.3), (2.3 ± 0.3), and (2.7 ± 0.5) × 10^–20^ m^2^/W, for the three wavelengths, respectively, and were the same for the five samples. These values and their dispersion are consistent with those reported in the literature, but their magnitude is about 27% lower than the average ones.

These findings suggest that the NER technique, particularly when combined with high-repetition-rate lasers, provides a robust approach for absolute nonlinear refractometry. This strategy can be extended to the characterization of emerging photonic materials that require an accurate determination of the nonlinear refractive index.

## Data Availability

The authors confirm that the data supporting the conclusions of this study are available in the article.
